# Supplementary material for: The causal relationship between the human gut microbiota and pyogenic arthritis: a Mendelian randomization study
Source: Front Cell Infect Microbiol. 2024 Nov 26;14:1452480. doi: 10.3389/fcimb.2024.1452480 (PMC11629706; doi:10.3389/fcimb.2024.1452480)
Supplement: Supplementary file 1 [file DataSheet1.zip › mendelian test/ebi-a-GCST90027847.csv_leave_one_out.pdf]

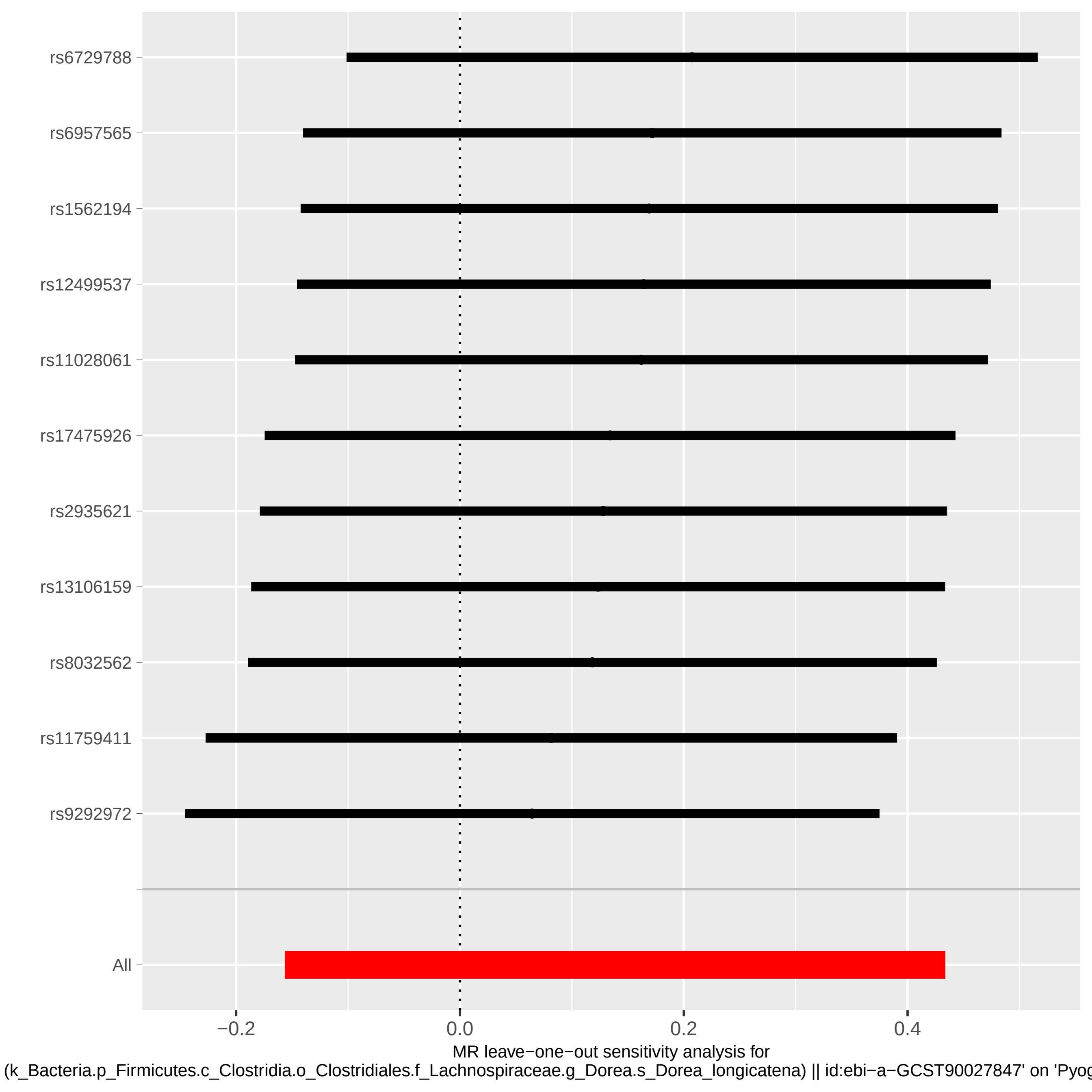

rs6729788
rs6957565
rs1562194
rs12499537
rs11028061
rs17475926
rs2935621
rs13106159
rs8032562
rs11759411
rs9292972
All
−0.2
0.0
0.2
0.4
MR leave−one−out sensitivity analysis for
(k_Bacteria.p_Firmicutes.c_Clostridia.o_Clostridiales.f_Lachnospiraceae.g_Dorea.s_Dorea_longicatena) || id:ebi−a−GCST90027847' on 'Pyog
